# On the Perceptive Power of the Spinal Chord, as Manifested by Cold-blooded Animals

**Published:** 1846-07

**Authors:** George Paton


### RECORD OF MEDICAL SCIENCE.

*On the perceptive power of the Spinal Chord, as manifested by cold-blooded animals.* By George Paton, M. D.—Physiologists have divided the movements of the animal economy into voluntary and involuntary. The former order comprises all those movements that are under our power and control; the latter, those that take place without our will, and independent of our agency. But a voluntary movement may be either of a spontaneous character, that is, it may originate in the mind from a particular train of ideas, and independent of any external stimulus operating to suggest it; or it may depend on an impression produced on the surface of the body, exciting the individual to the performance of the action, as when we withdraw a limb from any irritation to which it is exposed. But in both cases there is the utmost freedom in the movement, and every element essential to constitute it voluntary. In the one case, the mind is incited to the action by a train of thought connected with sensations and emotions which we had already felt and experienced. In the other, the stimulus, being immediately present to the senses, produces a feeling or emotion which leads to the performance of the action. It thus appears that the principle which constitutes an action voluntary is not the manner in which it is excited, but the mode in which it is performed,—a fact which will enable us more clearly to understand the nature of the movements performed by animals after removal of the cerebrum, and see how far such phenomena indicate sensation and the power of voluntary motion in an animal; or are to be accounted for, as maintained by some physiologists, on the principle of a reflex or excito-motory action of the spinal chord.

As this is a question which involves general principles of great importance in reference to the functions of the nervous system, we shall, in determining the nature of the phenomena observed, endeavour to show, according to the strict and philosophical import of the terms, that not merely sensation but perception enters into the character of the movements,—an indubitable proof that the animal can exercise control over its muscles, or has the power of regulating its movements to the attainment of a definite object.

"Sensation is a name given by philosophers to an act of mind, which may be distinguished from all others by this, that it hath no object distinct from the act itself." It is simply expressive of the pain or pleasure felt by the individual at the moment, but has no reference to the canse or irritation that produces it. When I say that I am suffering pain, I speak of a mere feeling or sensation of the mind, without relation to any external object or source from which the pain originates. And this is all that is to be understood by the term sensation. It is expressive of the state of mind, or manner in which I am affected at the moment, but nothing more,—in which sense, sensation and feeling are of exactly the same import.

But sensation is often employed, in a different sense, to signify the knowledge we have of an external impression by the sense of touch; and this meaning is more frequently attached to the term in accordance with the important discovery, that the spinal nerves consist of a sensory and a motory portion,—the former being employed to convey the impression to the medulla, and the latter to convey it from it, or to the parts to which it is distributed. As when we state that we consider an animal is possessed of sensation from the particular movements which it performs in response to a stimulus. But in strict and philosophical language, this use of the term implies not merely the sensation excited in the mind, but the knowledge and recognition of its cause, or the exercise of perception. Accordingly we find Mr. Stewart, in his Elements of Philosophy, drawing the distinction most accurately between these two principles. "If I strike my hand," says he, "against a hard object, I naturally say that I feel pain in my hand. The philosophical truth is, that I perceive the cause of the pain to be applied to that part of my body. The sensation itself I cannot refer in point of place to the hand without considering the mind to be spread over the body by diffusion." And the sentiments of Mr. Mill, one of the most recent writers on this subject, are of similar import. "When a stone lies before me I am conscious of certain sensations which I receive from it; but when I say these sensations come to me from an external object which I perceive, the meaning of these words is, that, receiving the sensations, I intuitively believe that an external cause of these sensations exists. Dr. Reid also, in his Essays on the Powers of the Human Mind, gives the same definition of the terms. "I shall conclude," says he, "by observing, that, as the confounding our sensations with that perception of external objects which is constantly conjoined with them, has been the occasion of most of the errors and false theories of philosophers with regard to the senses; so the distinguishing these operations seems to me to be the key that leads to the right understanding of both."

"Sensation taken by itself implies neither the conception nor belief of an external object. It supposes a sentient being, and a certain manner in which that being is affected, but it supposes no more. Perception implies an immediate conviction and belief of something external, something different both from the mind that perceives, and from the act of perception. Things so different in their nature ought to be distinguished, but by our constitution they are always united. Every different perception is conjoined with a sensation that is proper to it. The one is the sign, the other the thing signified."

Perception, then, may be defined to be that act of mind which consists in the recognition of an external object, or of an impression produced upon the surface of the body, or the extremities of the nerves by which the various sensations are excited in the mind. And in this sense every source of irritation distinctly recognised becomes an object of perception.

It will thus appear, if we attend to the nature of this operation of the mind so clearly stated by these philosophers, that in every irritation applied to the body there are two things that take place intimately connected, but nevertheless perfectly distinct; 1*st*, there is a sensation produced: 2*d*, there is a perception of its cause, or recoguition of the source from which it proceeds; and it is of the utmost importance to keep this in view in every inquiry of this nature, as it enables us to obtain a precise and definite idea of the nature of the phenomena observed, and to understand the real import and meaning of the terms employed to describe it.

It is also perfectly apparent, according to this definition of the subject, that in judging of the character of the movements of an animal, we must have evidence of perception in the movement before we can determine that the animal is possessed of sensation. For the particular movements of an animal are the signs by which its feelings are manifested to us, just as language is the sign or symbol which expresses the sentiments of man. It is because we observe an animal appear to take cognisance of the stimulus, and endeavour to avoid the irritation to which it is exposed, that we consider it is suffering pain. But in this there is the exercise of perception, or, as it has been termed, design and adaptation in the movement.

And in perfect accordance with these views of philosophers, physiologists recognise a distinct class of muscles, termed the muscles of voluntary motion, engaged in the performance of these movements, and of all those that manifest the will and intention of the animal; as the act of walking, leaping, running, the various and combined movements of the limbs, of the larynx, &c. These muscles are formed capable of a variety of movements, and rendered subservient to the will, evidently for the express purpose of making known our feelings, sentiments, and emotions, and every definite action performed by them must depend on the will and determination of the animal, as there is nothing in the structure of the parts to secure one particular movement in preference to every other. When we, therefore, witness design and adaptation of means to ends in movements performed by this class of muscles, as when an animal, on the application of a stimulus, raises its foot to the part of its body that is irritated, and endeavours to push away the instrument, it clearly indicates perception, or that the animal takes distinct cognisance of the stimulus, and as the movement is specific in its character, the principle on which it depends is not that the impression on the afferent nerve, on being conveyed to the medulla, is merely reflected by the afferent nerve, which constitutes reflex action. But here the impression produced on the afferent nerve gives rise to a distinct act on the part of the animal, by which, through the medium of the motor nerve, its feelings and perceptions are manifested to us. And there may be no immediate connection between the roots of these nerves or that which conveys the impression to the medulla, and that by which the movement is effected, as when an animal, on the integuments of its head being irritated, raises its hind foot to the part to remove the source of irritation.

On the other hand, in the involuntary movements of the body, as the acts of respiration, deglutition, sneezing, coughing, contraction of the iris, &c., there is no evidence of perception nor of any determinating power being exerted on the part of the animal, and consequently no proofs of sensation. For the parts are constructed in adaptation to the particular function which they perform and the action limited to the attainment of that end. Hence the movement always takes place in the same manner, without change or variation, and affords no evidence of design on the part of the animal, or of being subject to our power and control. Still there is evidence of design and adaptation of means to ends in these movements, as appears in every part of the beautiful structure of the animal economy; but it is design inherent in the structure of the part, and not dependent on the will of the animal. For the parts are so framed and constituted as to perform a specific function, by contracting on the application of a particular stimulus, and all that is necessary to excite these acts is, that each appropriate stimulus be applied to the extremities of the nerves, as light to cause contraction of the iris, &c. When we have a series of movements produced that are always performed in the same manner, each successive act being a repetition of that which preceded it. And the principle on which this class of movements depends is that of reflex action,—the impression on the afferent nerve, on being conveyed to the medulla, being immediately and independent of the will reflected by the afferent nerve, when a distinct set of muscles are thrown into action, and a specific function accomplished.

Hence in this class of movements, partaking of an involuntary and reflex character, the design being dependent on the structure of the part, gives no evidence of perception; and so far as these functions are observed in animals subjected to experiment, they could not enable us to determine the existence of sensation. But from what we feel in ourselves it is evident that several of these movements are essentially dependent on sensation, as the acts of sneezing and of coughing, so that it appears that several of the reflex movements of the body are intimately connected with the sensory tract of the spinal chord.

It would thus appear, 1*st*, that it is only in movements that are under the distinct control of the animal, performed by what are termed muscles of voluntary motion, that we can have any evidence of perception, as the involuntary and reflex movements give no indication of it; 2*d*, that the real and essential difference between a voluntary, and an involuntary, and reflex movement consists in the manner in which the motor nerve is excited, and not in the circumstance whether or not sensation may be produced by the stimulus applied to the incident nerve. For whilst we readily admit that many reflex movements take place totally unconnected with sensation, it is equally evident that in some most important movements of the animal economy, in which the action is distinctly reflex, sensation is excited by the irritation of the incident nerve, as in coughing, sneezing, &c. From which it would appear that these particular acts of reflex movement are connected with the sensory columns of the spinal chord.

II. From these preliminary observations, we shall proceed to inquire more particularly into the functions of the spinal chord; and ascertain if the phenomena manifested by animals after removal of the cerebrum or brain afford proofs of sensation and perception; or accord with the theory that the spinal chord is exclusively devoted to the functions of a reflex system of nerves, termed the true spinal system.

And in entering on this part of the inquiry, it will be proper to observe, that in physiology we cannot establish a general principle without the evidence of specific facts. In mathematics and natural philosophy we can arrive, from certain given data, with the greatest precision, at other ulterior truths; but in physiology, which is an inductive science, founded on observation and experiment, we cannot establish a general doctrine without having ascertained particulars. And this principle is the more necessary to be attended to, as it seems not unfrequently to have been lost sight of in researches into the structure and functions of the spinal chord.

We may also observe that, in eliciting the truth on this subject, we must reject every attempt to settle the question by any reasoning *a priori* concerning the nature of mental phenomena, as that a perceptive act must necessarily require the cerebrum for its development. This we need scarcely say is a *petitio principii* of the question, taking for granted the thing required to be proved. We can only determine the functions of the nervous system by the nature of the phenomena observed, for we must proceed in this, as in every inductive science, to found our views on a rigid induction of facts. And if these be clearly established, the doctrine must be admitted, as it rests on incontrovertible data. But if not, it must be considered a mere hypothesis destitute of proof.

How, then, do the facts ascertained, with regard to the functions of the nervous system, or the view at present maintained respecting the structure and functions of the spinal chord, correspond with the principles of inductive science? It is laid down by the supporters of an excito-motory system, that the spinal chord consists of two sets of nerves, one of which is connected with the central or cineritious portion of the chord, and ministers to the reflex movements of the body, termed the true spinal system, because it is considered to terminate in the spinal chord, and to have no connection with the cerebrum; and that there is another set of nerves connected with the cortical or medullary portion of the chord, and terminating in the cerebrum, which organ they consider the centre of this system of nerves, and the cause of all sensation, perception, and volition manifested by an animal. But this theory has not been confirmed by observations on the structure of the spinal chord. It has not been demonstrated that there is one set of nervous fibres connected with the cineritious portion of the chord, on which the reflex movements depend; and that a distinct set of fibres is connected with the medullary portion of the chord, and exclusively devoted to the senso-volitional movements of the body. And unless this be established, the theory cannot be considered as founded on the principles of inductive science.*

* "The microscope does not confirm the opinion of E. H. Weber, Bellingeri, and Grainger, that some fibres pass straightway to the central substance of the chord."—Mr. Paget's Report, Brit. and Foreign Med. Rev. Messrs.

But it is principally on physiological grounds that the theory has been supported,—on the movements observed in animals after removal of the cerebrum, or where the influence of the brain has been intercepted from a particular portion of the chord. But we maintain that these phenomena do not establish the theory of the spinal chord being exclusively devoted to an excito-motory system of nerves For animals, particularly of the cold-blooded class, after removal of the cerebrum, on being irritated, perform movements which afford the clearest proofs of perception, and, if we establish this fact, it will follow that there is a power resident in the spinal chord of taking cognisance of the stimulus, and performing regular and combined movements independent of the cerebrum.

The whole question then resolves itself into this, what is the state into which an animal is reduced, or the movements which it is capable of performing, after removal of the cerebrum? and the subject at present does not relate to the involuntary movements of respiration, sneezing, coughing, &c., of which we have already spoken, and admit to be of a reflex character; but to movements performed by what are termed muscles of voluntary motion, employed in walking, leaping, and the various movements of the limbs, &c., by which alone perception can be indicated.

We proceed then to show, that after removal of the cerebrum or brain, the phenomena manifested by an animal on the application of a stimulus indicate perception, or the power of regulating its movements to the attainment of an end.

*Experiment* I.—In a frog I removed the cerebral lobes with great care, and observed the phenomena.

On removal of the cerebral lobes the frog lost the power of spontaneous motion, as it scarcely moved except when irritated, but remained for weeks seated in a shallow vessel, amidst a little water, with its hind legs drawn up—the posture that frogs are accustomed to assume when they rest; and respiration remained perfect.

But on being irritated, it moved with great vigour, and gave every indication of recognizing the stimulus. When laid upon its back, it immediately turned upon its face, and then made several leaps before it became quiescent. On placing the finger over the spine in the interscapular region, it croaked distinctly. When seized by the foot, it struggled much to be relieved, and on being freed leapt to a distance. On irritating the integuments of its right dorsal region it raised its right hind foot and pushed away the instrument. And on irritating the integuments of its left shoulder, it immediately removed the instrument with its left hind foot. When I touched slightly with the point of a needle the integuments of its abdomen on the left side, it scratched the part with its left hind foot. And when I irritated the integuments of its lumbar region on the right side, it immediately raised up its right

Todd and Bowman also admit that the most recent observations on the structure of the spinal chord do not coincide with the views of Mr. Grainger.—Messrs. Todd and Bowman's Physiology, p. 331.

hind foot, and pushed away the instrument with force. On being raised up and then replaced upon the table, it would make several leaps before it became quiescent, and then draw up its hind legs, adjust itself, and assume the usual posture of rest; and did not again move till irritated.

Reflex movements could be produced in the extremities of this frog by compressing gently one of its toes, or irritating slightly with the point of a needle the integuments of its thorax. But the irritation always required to be performed with the greatest caution, and after the frog had remained quiescent for some time.

*Experiment* II.—In another frog I divided the *medulla oblongata* at the origin of the *trigemini* or fifth pair of nerves, and removed every portion of the encephalon anterior to this, and observed the phenomena. The effects of the operation had extended to the origin of the *par vagum*, as respiration continued for a little, but gradually ceased. The frog, however, survived the operation for several days, but no longer exhibited the power of spontaneous motion, as it did not move from the place where it was seated, till irritated; but on being irritated it gave the most indubitable proof of responding to the stimulus.

I compressed gently with the forceps one of the toes of its right hind foot, and it immediately withdrew it with great vigour, turning its body in the opposite direction. I irritated the integuments of its right shoulder, and it immediately scratched the part with its right hind foot. I then seized the part with the forceps and compressed it gently, when it raised up its right hind foot and pushed away the instrument. I touched slightly wlth the point of a needle the integuments of its left cervical region, and it quickly turned up its left fore foot to the part, and then moved forward its left hind foot, and scratched the part with it. I touched its cloaca with the point of a needle, and it crawled forwards a little. I surrounded it with objects to prevent its progressive motion, and then irritated its right dorsal region, when it immediately pushed away the instrument with its right hind foot.

I placed this frog upon its back, and irritated slightly with the point of a needle the integuments of its thorax in a longitudinal direction, and a reflex movement was observed in both its anterior extremities. I compressed gently one of the toes of its left hind foot, and a reflex movement was observed in the toes of that and the opposite extremity, and then the frog made an effort to raise itself up.

*Experiment* III.—In another frog I divided the *medulla oblongata* about the origin of the *par vagum*, and removed every portion of the brain, and observed the phenomena. Respiration ceased immediately.

On seizing with the forceps one of the toes of its right fore foot, it raised up its shoulder and endeavoured to withdraw its foot, but being retained it moved forward its right hind foot and planted it against the instrument, at the same time turning its body in the opposite direction, still endeavouring to extricate its foot. On irritating slightly its right dorsal region with the point of a needle, it raised its right hind foot, and pushed away the instrument. When I irritated the integuments of its abdomen on the left side, it immediately removed the instrument with its left hind foot, and then raised its foot and scratched the part with it. When I touched with the point of a needle the right lumbar region, it drew up the joint of its right hind leg over the part. When I pressed the left hind foot against the table with my finger, it struggled much to be relieved, and on being freed crawled to a considerable distance before it became quiescent. I then irritated its cloaca slightly with the point of a needle, and it drew up its hind legs over the part.

I placed this frog upon its back, and compressed gently with the forceps one of the toes of its right fore foot, and a slight reflex movement was observed in both its anterior extremities. I compressed gently one of the toes of its left hind foot, and a slight reflex movement was observed in all the toes of that extremity. On compressing the part more forcibly the limb was retracted, and a slight reflex movement was observed in the opposite extremity.

*Experiment* IV.—In a frog I divided the *medulla oblongata*, removing every portion of the brain. The division was made about the origin of the *par vagum* or eighth pair of nerves, which in the frog is the last pair of cerebral nerves. Respiration ceased immediately. After allowing the frog to remain at rest for a short time, I applied irritation, and observed the following phenomena.

On touching with the point of a needle the integuments over the left scapula, it raised its left hind foot, and pushed away the instrument. When I irritated the integuments of the right cervical region, it raised its right fore foot and scratched the part with it. When I irritated the integuments over the lower portion of the right scapula, it moved forwards its right hind foot and pushed away the instrument. On irritating the dorsal region the frog crawled forwards a little. On seizing with the forceps a toe of the right fore foot, it distinctly withdrew it. On the part being again seized and retained, it turned its body in the opposite direction, and endeavoured to withdraw its foot, and on being freed placed it below its thorax.

Reflex movements could be produced in this, as in other frogs, by a slight irritation of the extremities, and were always readily excited by irritating the integuments of its thorax when the frog was placed upon its back.

I performed the same experiment on many other frogs, and always with similar results; the animal after division of the *medulla oblongata* and complete removal of the brain, recognising most distinctly a stimulus applied to its body by raising up its foot and pushing away the instrument, or scratching the part that was irritated.*

From these experiments it appears that cold-blooded animals, after being deprived of the cerebrum or entire portion of the brain, can perform distinct perceptive movements, or evince cognisance of the stimulus applied to their body. And there must be sensation and distinct control over the muscles in these specific acts. For we maintain that no difference can be observed between these perceptive movements and those performed by unmutilated animals when subjected to similar irritation. The animal leaps, indeed, or performs regular progressive

* I may state, for the benefit of those who wish to verify these experiments, that they always succeed best early in spring, when the animal is taken, or has just emerged from its winter recess. In which case its physiological condition of a cold-blooded animal is particularly developed.

motion on the application of a stimulus—raises its limb, and removes with its foot the instrument that touches its body, or scratches the part that is irritated. In fine, it comports itself in every respect according to the degree and mode of irritation; and to deny that these movements are under the control of the animal, is to assert that it has no power over the voluntary muscles of its body when we witness in the movement the most distinct evidence of design. It is no doubt true that the animal ceases to exhibit the power of spontaneous motion, as its movements are performed chiefly in response to a stimulus. But then they are perfectly identical with the movements of an animal possessed of distinct voluntary power, and must consequently be performed by the same nerves, as no involuntary movement or principle of reflex action could produce such an effect.

Hence it appears that the function of the spinal chord in coldblooded-animals is not confined to the involuntary or reflex movements of respiration, deglutition, &c. But that we are to attribute to it an inherent power over the voluntary muscles of the body, in regulating and directing them to the attainment of a definite object, or performing distinct perceptive movements on the application of a stimulus.

And this doctrine, it will be observed, leads to a view of the function of the cerebrum opposed to that of M. Flourens, Dr. Marshall Hall and others, who consider that it is the seat of all the intellectual and perceptive powers of an animal.* But as we have indubitable proof that an animal may feel, perceive, and move in response to a stimulus, independently of the existence of the cerebrum, it appears that the functions of that organ is not to bestow these powers upon an animal, but to enable it to act on the possession of them, to remember its sensations and impressions, and render the knowledge thus acquired available to the purposes of life.†

* It will be observed that, whilst M. Flourens holds the theory of the excitability of the spinal chord, and Dr. Marshall Hall that of reflex action, they both agree in considering the cerebrum as necessary to an animal being possessed of sensation, and the power of perceiving an impression.

† The great difficulty with the opponents of this doctrine, that an animal is possessed of perception after removal of the cerebrum, is, that it shows no evidence of spontaneous motion and remembering the impression. But the absence of the one power is no proof against the positive existence of the other, when it is demonstrated by experiment. It surely does not follow that an infant is incapable of suffering pain, because its mental powers are still undeveloped? or that a person labouring under concussion of the brain does not feel an external impression, when he raises his hand to the part of his body that is irritated? or that the somnambulist does not walk or recognise an object, because they have no recollection of the act? The fact is, that the power of remembering an impression, so as to render it a subject of action, is totally distinct from the power of perceiving it. And whilst the former may be obscured or completely annihilated in an animal, the latter may remain and be distinctly manifested. A patient whom we visited, labouring under an affection of the head, with evident symptoms of pressure on the brain, lay for weeks in a state of deep stupor, insensible to every thing around her, and giving no indication of her wants except by a small movement of the lips, which her attendants observed when she required food or to be raised up. But on pinching her skin, she moaned or raised her hand to the part. It happened that during her illness she required the application of leeches to

2*d*. It will be observed, that, whilst we maintain the doctrine of the perceptive power of the spinal chord, we do not deny the principle of reflex action, though we consider that it may be connected with sensation, as all that is necessary to produce a reflex movement, is that the motor nerve be excited by the impression on the afferent nerve being communicated to it independent of the will of the animal; and this is the principle on which are constituted the involuntary or reflex movements of sneezing, coughing, &c. But we also admit that reflex movements may be excited in the voluntary muscles of an animal by any sudden and unexpected impression acting on the surface of the body or extremities of the nerves, as when a limb starts up involuntarily by being suddenly pricked with a pin, in which case the impression on the afferent nerve is immediately communicated to the motor nerve and excites the movement of the part. And we have seen that in a frog, after removal of the cerebrum or brain, reflex movements may be readily excited in the body and extremities of the animal by a slight irritation of the integuments or compression of a toe, &c. But these movements are always general and indefinite, and totally distinct from the perceptive movements performed by the same class of muscles.

From what has been already stated, it will be seen, that it is illogical to argue, as some physiologists have done, that as there is evidence of design and adaptation in the involuntary movements of the body, when the action is reflex, so the perceptive movements performed by an animal after removal of the cerebrum, may also be dependent on the same cause. For such a supposition, as we have already shown, is subversive of the data by which we determine the character of all perceptive movements. And we have only farther to add, that, as it may be laid down as a general proposition, that reflex action is of a general and indefinite nature, it could never effect a specific movement without a particular structure of the parts. When, therefore, it is maintained by the abettors of an excito-motory-system that a decapitated animal is reduced to a mere automatic state, totally incapable of sensation, and yet that movements perfectly indicative of design may be performed by the muscles of voluntary motion, on irritation being applied to the integuments, does it not appear that this amounts to nothing less than a contradiction in terms, or what mathematicians would call *reductio ad absurdum?*

The doctrines then which we deduce from these experiments are the following:—

1*st*. That the cerebrum is the seat of spontaneous motion, of memory and reflection.

2*d*. That the power of perceiving impressions, and performing movements in response to them, by a distinct control over the muscles her side, when her hands required to be held, to prevent her from removing them by force. And yet after her recovery, for she completely recovered, and is now in the enjoyment of good health, she declared that she remembered nothing of all that had transpired. But it would be difficult to suppose that she neither felt pain nor perceived the external impression.

of voluntary motion, is a property of the spinal chord, and possessed by an animal after removal of the cerebrum or brain.

*3d.* That the involuntary movements of respiration, deglutition, sneezing, coughing, defecation, &c., are dependent on a principle of reflex action possessed by the spinal chord.

*4th.* That the nerves connected with the muscles of voluntary motion may also take on reflex action, when an impression is produced suddenly on the surface of the body or extremities of an animal; but these movements are destitute of design, and easily distinguished from the perceptible movements performed by the same class of muscles.

In the experiments from which these doctrines have been deduced, it will be observed that every portion of the brain or encephalon anterior to the division of the *medulla oblongata* was removed, and that the division was often made so low as to affect the origin of the *par vagum*, which in the frog is the last pair of cerebral nerves; and yet the animal on being irritated gave the most distinct proofs of perceiving the impression and performing movements in accordance with it: and this we conceive is sufficient to set aside, so far as cold-blooded animals are concerned, the theory of senso-motory nerves being entirely connected with the cerebrum; or the proposition that in a decapitated animal no movements can be excited but those of reflex action.

We now proceed to consider the phenomena manifested by experiments on the salamander; and to show, that, on a still lower division of the chord, the animal possesses control over the movements of its lower extremities, and takes distinct cognisance of a stimulus.

In a salamander, (*Lacerta aquatica*, Linn.) I divided the spinal chord at the third spinal vertebra, and observed the phenomena. This division of the chord leaves the animal perfect power over its anterior extremities, as in the salamander the nerves going to the fore legs arise principally, as in the frog, from the second pair of spinal nerves.

After the effects of the operation had subsided, the animal raised itself upon its fore legs and began to move forwards, but did not drag its body like an animal that had suffered paralysis in its posterior extremities, but made distinct efforts with its hind feet in performing locomotion, supporting itself on them, as on its anterior extremities, and moving them alternately in accordance with the movements of its fore legs, as it crawled slowly along the surface of the table. And during these movements the divided ends of the chord did not come into contact, but remained distinct and separate.

I placed the animal upon its side, and it quickly turned upon its face, and its left hind leg remaining beneath its body, it distinctly withdrew it and began to move forwards, manifesting perfect power and control over its posterior extremities, but the movements were performed with less vigour than before the division of the chord.

When the animal after being quiescent again began to walk, spontaneous motion did not commence in its posterior extremities, but the animal raising itself on its fore legs began to move forwards, and then instead of dragging its hind feet, lifted them by turns, spreading out its toes as it supported itself on them, and moved slowly along the table.

After the animal remained at rest, I touched slightly, with the point of a needle, the integuments of the right dorsal region a little below the division of the chord, and it moved forwards its right hind foot and passed its toes distinctly across the part. I touched in a similar manner the integuments of the left dorsal region, and it quickly raised up its left hind leg, and scratched the part with its foot. On irritating the integuments on the right side of its abdomen, it raised its right hind foot to the part. On touching with the point of a needle a toe of its left hind foot, as it lay extended from its body, it raised its limb, and placed it along the side of the abdomen.

*Experiment* II.—In a salamander, I made a similar division of the chord, viz., at the third spinal vertebra, leaving the animal complete power over the movements of its fore legs, and then removed a small portion of the dorsal vertebræ and lower division of the chord, so that the divided ends could not come into contact.

Shortly after the operation the animal raised itself upon its fore legs, and began to walk, and moving slowly along the table did not drag its hind feet, but lifted up the one foot and moved it forwards, whilst it retained the other on the table, and so on in regular succession, in unison with the movements of its fore legs, as it performed locomotion. When placed upon its side, it turned upon its face, and withdrawing its right hind leg from beneath its body, again commenced to walk, and crawling over a scalpel which lay upon the table exerted its hind legs most distinctly in passing over the instrument; and during these movements the divided ends of the chord did not come into contact.

Whilst the animal lay quiescent, with its right hind leg stretched along the side of its tail, I touched gently with the point of a needle the lateral portion of its right dorsal region, a little below the division of the chord, and it immediately moved forward its right hind leg to the part. I touched it again, and it quickly raised its foot, curving its body still more than before, and passed its toes distinctly over the part. I irritated, in a similar manner, the lateral region of the opposite side of the body, and it raised up its left hind foot, and passed its toes most distinctly over the part. After a few minutes, I renewed the irritation, and it again raised up its foot, and passed its toes in a similar manner over the part. On touching with the point of a needle a toe of either of its hind feet, it raised the foot, and changed its position. During these movements its fore legs remained apparently quiescent.

*Experiment* III.—In a salamander, I made a similar division of the chord, viz., at the third spinal vertebra, leaving the animal perfect power over its forearms, and then removed a small portion from the lower division of the chord, so that the divided ends could not come into apposition, but remained separate, and observed all the phenomena manifested in the preceding experiments, both as regards the act of locomotion, and the various movements performed in response to a stimulus.

I then passed an instrument into the anterior portion of the spinal chord as far as the occiput, destroying completely that portion of the chord, and consequently all the movements of the anterior extremities.

And on touching with the point of a needle, the integuments of the left dorsal region immediately below the division of the chord, the animal quickly raised up its left hind foot, and scratched the part twice. I irritated in a similar manner, the integuments of the right dorsal region and the animal scratched the part with its right hind foot. On irritating the integuments on the right side of the abdomen, it raised up its right hind foot to the part. I again touched with the point of a needle the left dorsal region, and the animal raised up its left hind foot, and spread its toes over the part. On irritating the integuments of the head, lower jaw, &c., no movements could be produced in the lower extremities; showing that there was no nervous communication between the divided portions of the chord. But as soon as I touched the portion of the integuments supplied with nerves from below the division of the chord, the animal raised up its hind foot, and passed it over the part that was irritated. In short, it seemed to recognise most distinctly a stimulus applied below the division of the chord, but did not move without being irritated.

*Experiment* IV.—In a salamander, I divided the spinal chord immediately below the nerves going to the fore arms, allowing the animal to retain perfect power over its anterior extremities, and then removed a small portion of the lower division of the chord, rendering the part perfectly separate.

This animal, like the other salamanders, continued to possess the power of locomotion, manifesting control over the movements of its posterior extremities, as it crawled slowly along the table. On irritating the integuments below the division of the chord, it raised its hind leg, and scratched the part with its foot, and performed all the movements which we have described as manifested on a similar division of the chord.*

I now divided the spinal chord immediately behind the skull, laid open the immediate vertebræ, and removed that portion of the chord, viz., connected with the movements of its fore legs.

I then touched with the point of a needle the integuments of the right dorsal region immediately below the division of the chord, and the animal raised its right hind leg, and scratched the part with its foot, I irritated a similar portion of the left dorsal region, and the animal scratched the part with its left hind foot, and repeated the movement. On irritating the side of its abdomen with the point of a needle, it quickly raised its left hind foot to the part, and on touching the right side of its abdomen in a similar manner, it immediately raised its right hind foot, and passed its toes most distinctly over the part. When I irritated the integuments of either foot slightly with the point of a needle, it quickly raised it up and changed its position.

* Most of these experiments were performed in spring, when the animal had just left its winter quarters; and from the physiological condition of the animal, the experiments always succeed best at that period of the year. And many of them were witnessed by competent judges, who can attest the accuracy of the operation and phenomena observed.

During this period no movements could be excited in the lower extremities by irritating the integuments of the head, lower jaw, &c., showing that there was no communication between the divided portions of the chord. But on touching with the point of a needle the integuments of either side of the body immediately below the division of the chord, the animal quickly raised up its hind foot, and scratched the part with it in the manner described—thus recognising most distinctly a stimulus applied below the division of the chord.

*Experiment* V.—In a salamander, I made a similar division of the chord, viz., at the third spinal vertebra, and observed the phenomena which we have described as manifested by the other salamanders—the animal retaining power over its posterior extremities, so as to assist in the act of locomotion; and recognising most distinctly a stimulus applied below the division of the chord, as it raised its hind foot, and scratched the part of its body that was irritated, &c.

A few days after this, I divided the spine immediately behind the skull, and removed the portion of chord contained in the intermediate vertebræ, and also passed an instrument into the cranium, and completely removed the encephalon.

I now irritated with the point of a needle the integuments of the right dorsal region immediately below the division of the chord, and the animal raised its right hind leg, and scratched the part distinctly with its foot. I irritated in a similar manner the integuments of the left dorsal region, and it scratched the part with its left hind foot. I touched with a needle the integuments of the right side of its abdomen, and it raised its right hind foot to the part. On irritating the toe of either of its hind feet, it quickly raised its foot, and placed it in a different position. I irritated the integuments of the left dorsal region, and it raised its left hind foot and spread its toes over the part. But it did not move without being irritated.

I performed the same experiment on many other salamanders, and with similar results, the animal, after the encephalon and upper portion of the chord had been removed, recognising the stimulus applied to the integuments supplied with nerves from below the division of the chord. But in these cases, an interval was often required to elapse before the irritation was renewed, in order that the movements might be distinctly manifested.

1st. These experiments, it will be observed, exhibit very remarkable phenomena. But still, as far as regards the function of the spinal chord, these phenomena are of exactly the same nature as witnessed by experiments on the frog, the only difference being that, in the former case, the chord was divided anterior to the origin of the *par vagum*, and the whole of the brain removed; but in this the chord was divided a little lower in the spine, viz., below the second pair of spinal nerves, and all communication intercepted from the cerebral lobes, or they were entirely removed, so that, as far as relates to the functions of the spinal chord, after it is cut off from all influence and communication with the brain, the movements are essentially the same in character.

2d. The phenomena manifested by these experiments, cannot, we maintain, be accounted for on the principle of reflex action. For the elements of a controlling power or agency which enters into these movements renders them incapable of being explained by the impression on the afferent nerve, being merely reflected by the efferent or motor nerve of the part. And in all the examples of reflex action that have been witnessed in man, or described as occurring in warm-blooded animals after division or injury of the spinal chord, the movements have been of a general and indefinite character; the muscles of the inferior extremities merely contracting on the application of a stimulus, so as to produce extension and then retraction of the limb, but nothing like a definite power being manifested. In a case of reflex action which came under our own observation, and to which we particularly attended, the patient laboured under Pott's disease of the last dorsal vertebra, with complete loss of sensation and power of voluntary motion in his inferior extremities. On irritating slightly the toes of either foot, that or even both limbs were immediately retracted and drawn up towards the abdomen, and maintained a vibratory motion for some time, which was always renewed on applying the irritation. But no appearance of control was manifested in these movements, as in the acts of locomotion performed by the salamander. And if the one class of movements be admitted to be of a reflex character, then the other must be considered to be specifically different, as elements of a controlling agency are manifested. Even M. Flourens, who holds the theory of the excitability of the spinal chord, acknowledges that the salamander has the power of moving its inferior extremities and performing acts of locomotion, after the division of the chord; a phenomena which is not observed in warm-blooded animals.*

* "Si, sur un animal a sang chaud, sur un oiseau, sur un mammifère, par exemple, on divise un point quelconque de la moelle épinière par une section transversale, aussitôt toutes les parties situées au-dessous du point divisé sont frappées de paralysie. Si, par exemple, la section a été faite au-dessus du renflement posterieur de la moelle épinière, aussitot les jambes de derrière sont frappees de paralysie; l'animal les traine, mais il ne les meut plus.

"Il n'en est pas ainsi pour les salamandres. L'animal continue a mouvoir ses jambes et sa queue, quoique la moelle épinière, et meme toute la colonne vertebrale, soient coupees fort au dessus de l'origine des nerves des jambes et de la queue.

"Je coupai la colonne vertebrale, et la moelle épinière, sur une salamandre.

"Immediatement apres l'operation, l'animal remuait deja ses pattes de derriere, et sa queue.

"Un mois plus tard, il les remuait, ou les mouvait beaucop mieux encore. Il marchait, et faisait avancer tour a tour, pour marcher, chaque jambe de derriere, comme il faisait avancer tour a tour celles de devant.

"Cependant le reunion des deux bouts de moelle épinière divises n'avait point en lieu.

"J'ai repete cette experience sur plusieurs autres salamandres.

"Elles ont toutes survecu pendant plusieurs mois.

"Et, de plus, elles remuaient toutes de meme, et surtout quelques mois apres l'operation, leurs jambes de dérriere, et leur queue."—Recherches Experimentales sur les Proprietes et les Functions du Systeme Nerveux, dans les Animaux Vertebres. Par P. Flourens, p. 420.

*3d.* We maintain that these phenomena afford distinct evidence of the perceptive power of the spinal chord. The animal, we have shown, does not merely retain the power of moving its limbs, but of moving them with order and regularity, and to the attainment of a specific end, taking distinct cognisance of the stimulus applied below the division of the chord, by raising up its hind leg, and scratching with its foot the part of its body that is irritated. And these movements it performs not merely after the chord has been divided, but after the upper portion has been removed as far as the occiput, so that no influence could be communicated from the brain to that segment of the chord,—nay, after the encephalon has been destroyed. And if these movements be not of a perceptive character, then there is no meaning in language, and we must give a new definition to the term perception. For it is of no avail to urge against these facts, the frequently repeated objection, that many movements of adaptation depend on reflex action, and that these may be of an analogous nature. As well may it be asserted, that the perceptive movements performed by an animal before division of the chord are of a reflex character. as that these belong to that category.

*4th.* But perhaps it may be stated, as a metaphysical objection, that it seems opposed to the unity of mind, for a perceptive power to be manifested by the spinal chord, when it is cut off from all influence and communication with the brain. But no metaphysical theory can ever set aside a positive fact, which we maintain is established by these experiments. We must interpret the laws of nature according to the phenomena observed; and if it can be shown on the principles of inductive science—by clear and distinct experiment—that an animal is capable of recognising a stimulus by the power possessed by the spinal chord, when it is cut off from all communication with the brain, then, to overturn this opinion, it will be necessary to disprove the fact, and this we maintain is impossible.—*Edinburgh Med. and Surg. Journ.*

